# The effect and safety of small-molecule antiviral drugs on long prognosis of post-COVID-19 pulmonary fibrosis: a real-world study in China

**DOI:** 10.3389/fmicb.2025.1733465

**Published:** 2026-01-06

**Authors:** Yilin Xu, Ming Lu, Yawei Li, Xiaoling Den, Dechang Peng, Fuqing Zhou, Anqi Li, Tieying Hou, Tianxin Xiang

**Affiliations:** 1 Infection Control and Prevention Center, The First Affiliated Hospital, Jiangxi Medical College, Nanchang University, Nanchang, China; 2 Jiangxi Provincial Key Laboratory of Prevention and Treatment of Infectious Diseases, Nanchang, China; 3 China-Japan Friendship Jiangxi Hospital, National Regional Center for Respiratory Medicine, Nanchang, China; 4 Jiangxi Medical Center for Critical Public Health Events, Nanchang, China; 5 Medical Imaging Center, The First Affiliated Hospital, Jiangxi Medical College, Nanchang University, Nanchang, China; 6 Medical Experimental Center, Shenzhen Nanshan People's Hospital, The 6th Affiliated Hospital of Shenzhen University Medical School, Shenzhen, China

**Keywords:** COVID-19, pulmonary fibrosis, small-molecule antiviral drug, post-COVID-19 pulmonary fibrosis, prognosis

## Abstract

**Background:**

Post-COVID-19 pulmonary fibrosis (PCPF) is one of the most common diagnoses after COVID-19 acute infection. However, the interventions for long-term prognosis of PCPF are relatively lacking.

**Aim:**

We aimed to observe one-year readmission and mortality rates among patients with PCPF, and tried to explore the impact of small-molecule antiviral drugs (SMADs) during hospitalization on the prognosis.

**Method:**

A total of 372 patients diagnosed as PCPF were enrolled. 293 of them treated with SMADs and the remaining 79 treated with NO SMADs. One-year readmission and mortality rates were evaluated via regression analysis.

**Results:**

During hospitalization, 16 of the 372 patients died. An additional 19 survivors died within one-year postdischarge. Among survivors, 49.62% were readmitted. Compared to the NO-SMAD group, the SMAD group presented a lower one-year readmission rate (45.97%vs. 63.64%, *p* = 0.020) and a reduced risk of one-year readmission (HR = 0.65, 95% CI: 0.44 to 0.96, *p* = 0.030).

**Conclusion:**

Nearly half of PCPF patients experienced readmission within one-year following their initial hospitalization for acute COVID-19. Importantly, treatment with SMADs during the acute infection phase was significantly associated with a reduced readmission rate.

## Introduction

The management of persistent discomfort after acute coronavirus disease 2019 (COVID-19) has emerged as a primary concern. The resulting pulmonary fibrosis is one of the most common post-COVID-19 diagnoses (Babar et al., 2024), also named post-COVID-19 pulmonary fibrosis (PCPF) (Ambardar et al., 2021; Kostopanagiotou et al., 2022). Extensive clinical research has been performed in the last few years to explore mechanisms or risk factors associated with PCPF. The identification of risk factors for PCPF is consistent; however, the incidence rates have fluctuated as a consequence of the evolution of the SARS-CoV-2 virus and variations among research institutions (Tanni et al., 2021; Bocchino et al., 2023). Therefore, constant attention to the long-term prognosis of patients with PCPF is essential, given the substantial number of affected patients and the perpetual evolution of SARS-CoV-2.

Prior studies have classified post-COVID-19 pulmonary CT results into three categories: (1) predominantly ground glass; (2) mixed ground glass and fibrotic; and (3) predominantly fibrotic (Solomon et al., 2021). The overlap of the pulmonary alveolitis phase and fibrosis phase, along with the lack of direct indicators, complicates diagnosis, unless a patient is monitored with multiple CT scans and a further understanding of PCPF, seriously delaying treatment. Consequently, timely, convenient, and effective treatment strategies are urgently needed.

Anti-inflammatory and antifibrotic therapies have emerged as focal points in PCPF research given the critical roles of inflammation and fibrosis in this process (Myall et al., 2021; George et al., 2020; Kooistra et al., 2023; Chen et al., 2022). However, the clinical efficacy of these therapies remains a subject of debate or is largely theoretical (Kooistra et al., 2023). As investigations into long-term COVID-19 progress, reports have been published on the extensive impacts of the persistent reservoir of SARS-CoV-2 on various organs, highlighting the necessity of excluding residual effects induced by the virus (Yao et al., 2023). The significance of small-molecule antiviral drugs (SMADs) during the viral infection phase in affecting PCPF outcomes has gradually been recognized, although it has yet to be thoroughly documented.

Accordingly, we ensured the inclusion eligibility of all the enrolled patients who were identified as having PCPF following strict inclusion and exclusion criteria. We aimed to further explore the one-year prognosis, especially the readmission rate of survivors, which represents a significant financial burden given the high prevalence of infectious cases. Moreover, we aimed to determine the effect of SMADs on the prognosis of patients with PCPF. This could provide an important contribution to the expanding research on the relationship between antiviral therapy and the prognosis of patients with PCPF.

## Methods

### Study design

This was a real-world study of patients who were diagnosed with PCPF. All the individuals who received a definitive diagnosis of COVID-19 during their inpatient stay at the designated medical institution designated for COVID-19 treatment between December 1, 2022 to January 31, 2023 were included in the preliminary screening. Following the comprehensive assessment of chest CT images (± 1 month around the acute infective phase) and relevant medical records from our institution, anyone with symptoms underwent further evaluation. Inclusion Criteria: ① Laboratory-confirmed SARS-CoV-2 infection within 30 days, supported by a positive antigen or nucleic acid test from medical records or official reports. ② Eligible patients must have two or more evaluable chest CT scans, (a) at least one scan performed during acute-phase hospitalization, paired with another conducted within 1 month prior to admission or within 1 month after discharge or (b) any two scans acquired more than 3 days apart during the index hospitalization. For all patients meeting these criteria, every available CT image obtained during the observation period was collected and included in the analysis. ③ Chest CT scan demonstrating abnormalities consistent with fibrotic change (The fibrotic lesions defined in “Chest CT Examinations and Quantification” section). Exclusion Criteria: ① Patients lacking complete and continuous medical records required for the study, including documentation of symptoms, medication use, and pre- and post-treatment laboratory results. ② Age≤18 years. ③ Absence of change in fibrotic lesions. ④ The change of fibrotic lesions determined by the research team to be attributable to trauma, surgery, or other pathogens. Ultimately, 372 patients identified with a definite diagnosis of PCPF were included for subsequent follow-up (Supplementary Figure 1).

### Clinical data collection

#### Hospital stay

Comprehensive data, including age, sex, department, diagnosis and dates of admission and discharge, were collected for all hospitalized patients with SARS-CoV-2 infection. Following the identification of PCPF, COVID-19-related symptoms and vital signs were collected upon admission. Information about oxygen therapy and COVID-19 treatments, including SMADs, glucocorticoids, immunoglobulins and antibiotics, was subsequently collected during hospitalization. To obtain a thorough understanding of the clinical situation and outcomes of patients following treatment, inflammation-related laboratory parameters as well as chest CT scans obtained before and after treatment were extracted from the patients’ medical records. The inflammation-related laboratory parameters included lymphocytes (10^9^/L) and C-reactive protein (CRP) (mg/L) (Myall et al., 2021). The therapeutic efficacy was assessed on the basis of the evaluation of symptoms and examinations conducted before and after treatment. Finally, data about in-hospital mortality were extracted from discharge notes and death summaries.

#### One-year follow-up

The surviving PCPF patients were followed up for one year after discharge. Follow-up was performed via outpatient or inpatient visits. The one-year outcome variables included ① the cause and date of death, and ② the cause and date of first hospital readmission. Patients with the wrong phone number or who refused follow-up visits were excluded.

### Antiviral therapy with small-molecule drugs

The antiviral therapies that were used to treat COVID-19 during the acute phase and were included in this analysis consisted of small-molecule drugs (nirmatrelvir/ritonavir). All medications were orally administered as advised by physicians, following recommended dosages and considering potential drug interactions. Data regarding SMADs were obtained from medical records, medication orders within this tertiary care facility, or purchase records provided by patients. Two physicians (an infectious disease specialist and a respiratory disease specialist) completed and verified the medication data independently.

### Chest CT examinations and quantification

All the chest CT examinations were conducted in the provincial designated medical institution. Two radiologists with experience in chest radiology reviewed the images independently. Any combination of ground–glass opacities (GGOs), reticulation, bronchiectasis, and/or honeycombing was considered to indicate fibrosis (Babar et al., 2024). The criteria for determining COVID-19-related fibrosis included the following: (1) aggravated fibrotic lesions during hospitalization compared with those observed 1 month prior to admission; (2) progressive aggravation of fibrotic lesions during hospitalization; and (3) increased severity of fibrotic lesions within 1 month after discharge compared with those noted during hospitalization. Notably, the localized changes resulting from trauma or surgery, as well as changes due to other pathogens diagnosed by respiratory physicians, were excluded from the final PCPF analysis. Four doctors (two radiologists and two respiratory physicians) utilized the Ichikado CT score for quantification (Supplementary Figure 2), and this score has been demonstrated to be highly sensitive for detecting pulmonary histopathological fibrosis in PCPF patients (Ball et al., 2021). The final score was averaged from the assessments made by all four doctors.

### Statistical analysis

Continuous variables were tested for a normal distribution via the Kolmogorov–Smirnov test and then expressed as medians (interquartile ranges [IQRs]). The Mann–Whitney test was used for single comparisons, whereas the Kruskal–Wallis test was used for multiple comparisons. Categorical variables are presented as counts (*n*) and percentages (%) and were compared via the χ^2^ test. Linear regression (continuous outcomes) and logistic regression (dichotomous outcomes) were performed to assess the associations between SMADs treatment and outcomes (readmission and death). For survival analysis, the hazard ratio (HR) was calculated via univariate Cox regression analysis. All the analyses were performed using SPSS software (SPSS version 25) and R Software (4.2.1). The R software packages used included ggplot2, stats, car, and survival.

## Results

**Figure 1:**
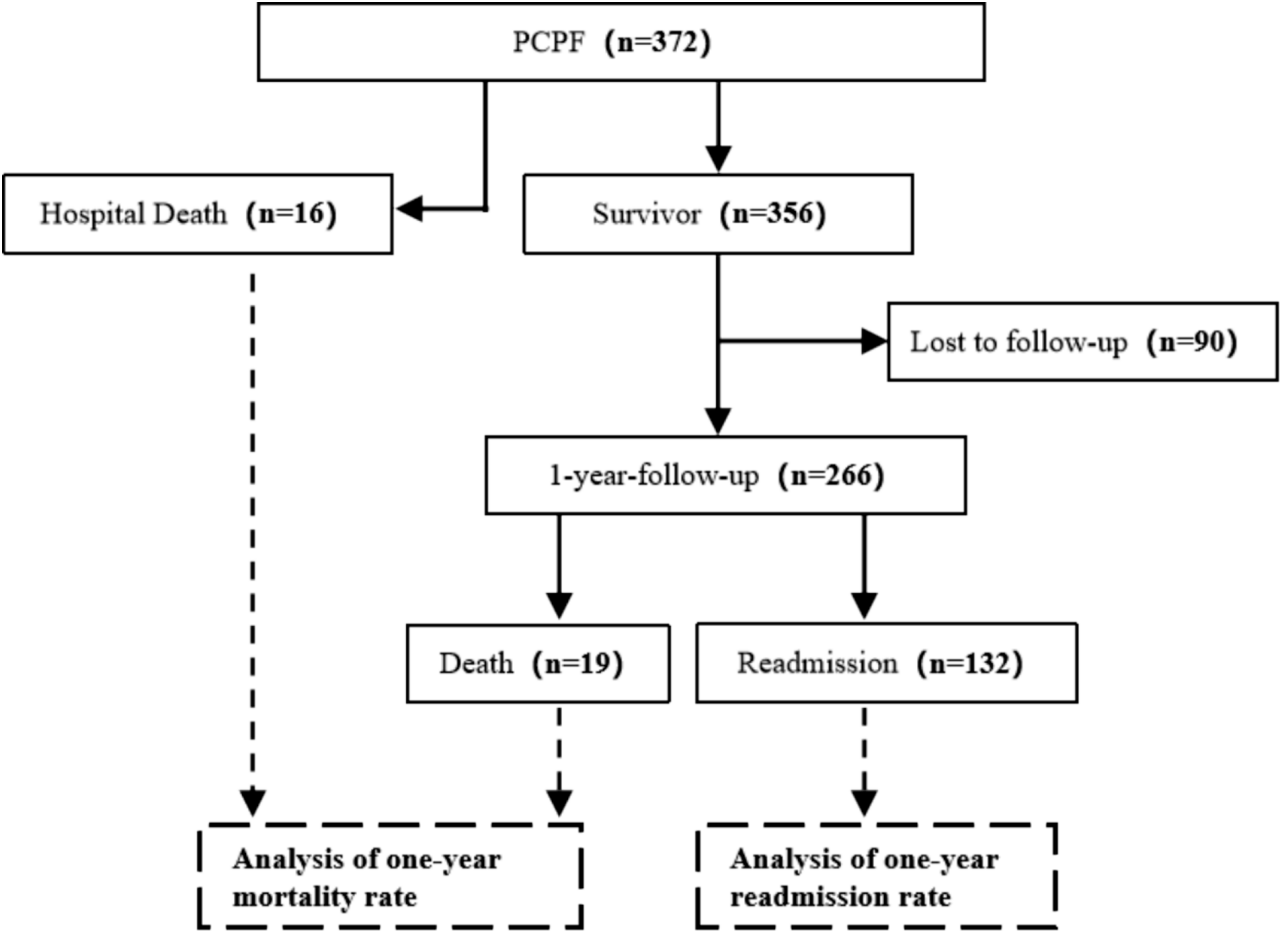
Flowchart of the study population. PCPF, post-COVID-19 pulmonary fibrosis; COVID-19, Coronavirus disease 2019.

A total of 11,733 adult patients with SARS-CoV-2 were admitted during the study period. Among these patients, 4,925 patients had severe disease and required admission to the ICU or suffered from severe respiratory failure or shock while hospitalized in a general ward. Based on the comprehensive review of chest CT scans and medical records of all the patients, 372 (3.17%) patients diagnosed with PCPF were included in the final analysis. Ultimately, PCPF was identified in 2.78% (137/4,925) of patients with severe disease (Supplementary Figure 1). 90 patients were lost to follow-up after discharge. Consequently, follow-up records were available for 266 patients. A total of 132 patients were readmitted within one-year after discharge. Sixteen PCPF patients died during the hospital stay (4.30%), whereas an additional nineteen died within one-year following discharge. A total mortality rate of 12.41% (35/282) was observed among patients with observational outcomes throughout the study period (Figure 1).

### Characteristics of PCPF patients

Briefly, the PCPF patients in this study were predominantly older (median 71 years) and male (65.32%). The cohort exhibited no significant obesity issues, as indicated by a median BMI of 22 (95% CI: 20.82–24.83). Few participants were current smokers (11.02%) or consumers of alcohol (7.53%). Most had at least one comorbidity, with the most common being hypertension (52.15%) and diabetes (29.03%). Additionally, sixty-six participants (17.74%) received some form of immunosuppressive therapy (glucocorticoids, other drugs, or both). Further details on other comorbidities are provided in Table 1.

**Table 1:** Characters of PCPF patients.

Variables	All PCPF	NO SMAD	SMAD	*p*-value
	*N* = 372	*N* = 79	*N* = 293	
Age (year)	71 (59–80)	69 (60–79)	72 (59–81)	0.326
Gender				
Female	129 (34.68%)	32 (40.51%)	97 (33.11%)	0.220
Male	243 (65.32%)	47 (59.49%)	196 (66.89%)	
BMI	22.86 (20.82–24.83)	22.15 (20.54–24.27)	22.92 (20.96–24.91)	0.093
Current smoker	41 (11.02%)	6 (7.59%)	35 (11.95%)	0.273
Current drinker	28 (7.53%)	6 (7.59%)	22 (7.51%)	0.979
Comorbidity				
Hypertension	194 (52.15%)	38 (48.10%)	156 (53.24%)	0.417
CVD	79 (21.24%)	22 (27.85%)	57 (19.45%)	0.105
COPD	54 (14.52%)	10 (12.66%)	10 (12.66%)	0.597
TB	18 (4.84%)	4 (5.06%)	14 (4.78%)	0.917
Diabetes	108 (29.03%)	19 (24.05%)	89 (30.38%)	0.272
Hyperlipidemia	18 (4.84%)	2 (2.53%)	16 (5.46%)	0.282
CLD	41 (11.02%)	13 (16.46%)	28 (9.56%)	0.082
CKD	68 (18.28%)	16 (20.25%)	52 (17.75%)	0.609
Rheumatic	36 (9.68%)	10 (12.66%)	26 (8.87%)	0.313
Anemia	85 (22.85%)	26 (32.91%)	59 (20.14%)	0.016
Cancer	79 (21.24%)	19 (24.05%)	60 (20.48%)	0.491
Medication^a^	66 (17.74%)	13 (16.46%)	53 (18.09%)	0.736
Severe	137 (36.83%)	31 (39.24%)	106 (36.18%)	0.616
Vital signs				
Temperature (°C)	36.60 (36.40–37.10)	36.50 (36.20–37.00)	36.65 (36.50–37.20)	0.069
SBP (mmHg)	127 (115–140)	125 (116–140)	127 (115–140)	0.587
DBP (mmHg)	74 (67–82)	76 (67–82)	74 (67–83)	0.925
Pulse (per min)	89 (78–100)	91 (81–101)	88 (78–100)	0.368
Symptoms of COVID-19				
No fever	78 (20.97%)	23 (29.11%)	55 (18.77%)	0.085
Low fever (37.2–38.5)	154 (41.40%)	26 (32.91%)	128 (43.69%)	
High fever (>38.5)	140 (37.63%)	30 (37.97%)	110 (37.54%)	
Fatigue	137 (36.83%)	35 (44.30%)	102 (34.81%)	0.121
Cough	343 (92.20%)	66 (83.54%)	277 (94.54%)	0.001
Sputum	326 (87.63%)	61 (77.22%)	265 (90.44%)	0.002
Nasal congestion/runny nose	13 (3.49%)	3 (3.80%)	10 (3.41%)	0.869
Sore throat	35 (9.41%)	7 (8.86%)	28 (9.56%)	0.851
Digestive	20 (5.38%)	5 (6.33%)	15 (5.12%)	0.672
Myalgia	34 (9.14%)	8 (10.13%)	26 (8.87%)	0.732
Chest distress	156 (41.94%)	27 (34.18%)	129 (44.03%)	0.115
Dyspnea	86 (23.12%)	21 (26.58%)	65 (22.18%)	0.411
Oxygen therapy (>24 h)				
No oxygen therapy	49 (13.17%)	20 (25.32%)	29 (9.90%)	<0.001
Non invasive oxygen therapy	305 (81.99%)	53 (67.09%)	252 (86.01%)	
Invasive oxygen therapy	18 (4.84%)	6 (7.59%)	12 (4.10%)	
Glucocorticoid				
Glucocorticoid treatment	327 (87.90%)	58 (73.42%)	269 (91.81%)	<0.001
Treatment began on admission^b^	231 (70.64%)	34 (58.62%)	197 (73.23%)	<0.001
Duration of treatment (day)	11 (7–16)	10 (7–16)	11 (8–16)	0.323
Immunoglobulin				
Immunoglobulin treatment	84 (22.58%)	11 (13.92%)	73 (24.91%)	0.038
Treatment began on admission^b^	31 (38.10%)	4 (36.36%)	27 (36.99%)	0.751
Duration of treatment (day)	4 (2–5)	4 (3–5)	3 (2–5)	0.310
Antibiotic				
No antibiotic treatment	20 (5.38%)	6 (7.59%)	14 (4.78%)	0.363
Monotherapy	149 (40.05%)	27 (34.18%)	122 (41.64%)	
Combination therapy	203 (54.57%)	46 (58.23%)	157 (53.58%)	
Duration of treatment (day)	14 (10–19)	15 (11–22)	14 (10–19)	0.270
Hospitalization				
Hospital death	16 (4.30%)	7 (8.86%)	9 (3.07%)	0.024
Hospital stay (day)	15 (11–21)	15 (11–22)	15 (11–20)	0.512
One-year-follow-up				
Patients with One-year-follow-up	266 (71.51%)	55 (69.62%)	211 (72.01%)	0.715
Readmitted^c^	132 (49.62%)	35 (63.64%)	97 (45.97%)	0.020
Readmitted day (day)	77 (23–204)	74 (29–189)	77 (19–204)	0.869
One-year death^d^	35 (12.41%)	12 (19.35%)	23 (10.45%)	0.060
One-year death day (day)	28 (0–184)	0 (0–197)	67 (0–175)	0.648

a Immunosuppressant or corticosteroids.

b The percentage = 100% × number of treatment begun on admission/number of total treatment.

c The percentage = 100% × number of Readmitted/Patients with One-year-follow-up.

d *N* = One-year death after discharge of follow-up patients + hospital death. The percentage = 100% × number of one-year death/(Patients with One-year-follow-up + hospital death).

PCPF, post-COVID-19 pulmonary fibrosis; COVID-19, coronavirus disease 2019; SMAD, small molecule antiviral drug; BMI, body mass index; ILD, interstitial lung disease; CVD, cardiovascular disease; COPD, chronic obstructive pulmonary disease; TB, tuberculosis; CLD, chronic liver disease; CKD, chronic kidney disease.

We investigated antiviral medication use retrospectively, and the results revealed that 79 (21.24%) patients did not receive any SMAD treatment during acute COVID-19 disease. No significant differences were observed regarding age, sex, BMI, or comorbidities between the SMAD and NO-SMAD groups except for anemia (*p* = 0.016) (Table 1).

Although most patients presented with normal vital signs when admitted, a total of 136 (22.8%) patients were admitted critically ill. During hospitalization, 78 (20.97%) patients did not experience fever. Among the remaining patients, 154 (41.40%) experienced low fever (37.2 to 38.5 °C), whereas 140 (37.63%) experienced high fever (>38.5 °C). The incidence rates of fatigue, cough, sputum, chest distress, and dyspnea were 36.83, 92.20, 87.63, 41.94, and 3.12%, respectively. Few patients suffered from nasal congestion/runny nose (3.49%), sore throat (9.41%), digestive (5.38%), or myalgia (9.14%). No patient reported impairment in smell or taste, and none reported conjunctivitis. Compared with the SMAD group, the NO-SMAD group presented significantly lower rates of cough (83.54% vs. 94.54%, *p* = 0.001) and sputum (77.22% vs. 90.44%, *p* = 0.002). The baseline CT score upon admission was 123.33 (95% CI: 110.00–139.38), and no significant differences were found between the SMAD and NO-SMAD groups (Table 1).

### Therapy and response to therapy

In response to hypoxemia, oxygen therapy was provided for the majority of patients, including noninvasive oxygen therapy (e.g., nasal catheter oxygen therapy, mask oxygen therapy, noninvasive ventilation, high-flow nasal cannula oxygen therapy and noninvasive mechanical ventilation) (81.99%) and invasive ventilation (4.84%). Among the 372 patients, 327 (87.90%) received glucocorticoid therapy for a median of 11 (7–16) days, with 70.64% starting on the day they were admitted; 84 (22.58%) received immunoglobulin treatment for a median of 4 (2–5) days, 38.10% of whom began on the day of admission. A large proportion of patients in our study were prescribed antibiotics for pulmonary infections (94.62%), with a median treatment course of 14 days, 40.05% for monotherapy and 54.57% for combination therapy. Compared with the NO-SMAD group, the SMAD group received significantly more oxygen therapy (74.68% vs. 90.11%, *p* < 0.001), glucocorticoids (73.42% vs. 91.81%, *p* < 0.001) and immunoglobulins (13.92% vs. 24.91%, *p* = 0.038) (Table 1).

Blood markers indicated improved systemic inflammation at discharge, with lymphocytes increasing from a median of 0.76 (0.53–1.16) × 10^9^/L at admission to 1.00 (0.66–1.42) × 10^9^/L at discharge (*p* < 0.001) and CRP decreasing from a median of 31.14 (10.84–72.72) mg/L at admission to 9.28 (2.33–25.10) mg/L at discharge (*p* < 0.001). The same significantly different trends were observed in the abnormal cases of lymphocytes (71.77% vs. 55.11%, *p* < 0.001) and CRP (75.81% vs. 53.26%, *p* < 0.001) (Supplementary Table 1).

### Chest CT scan findings

**Figure 2:**
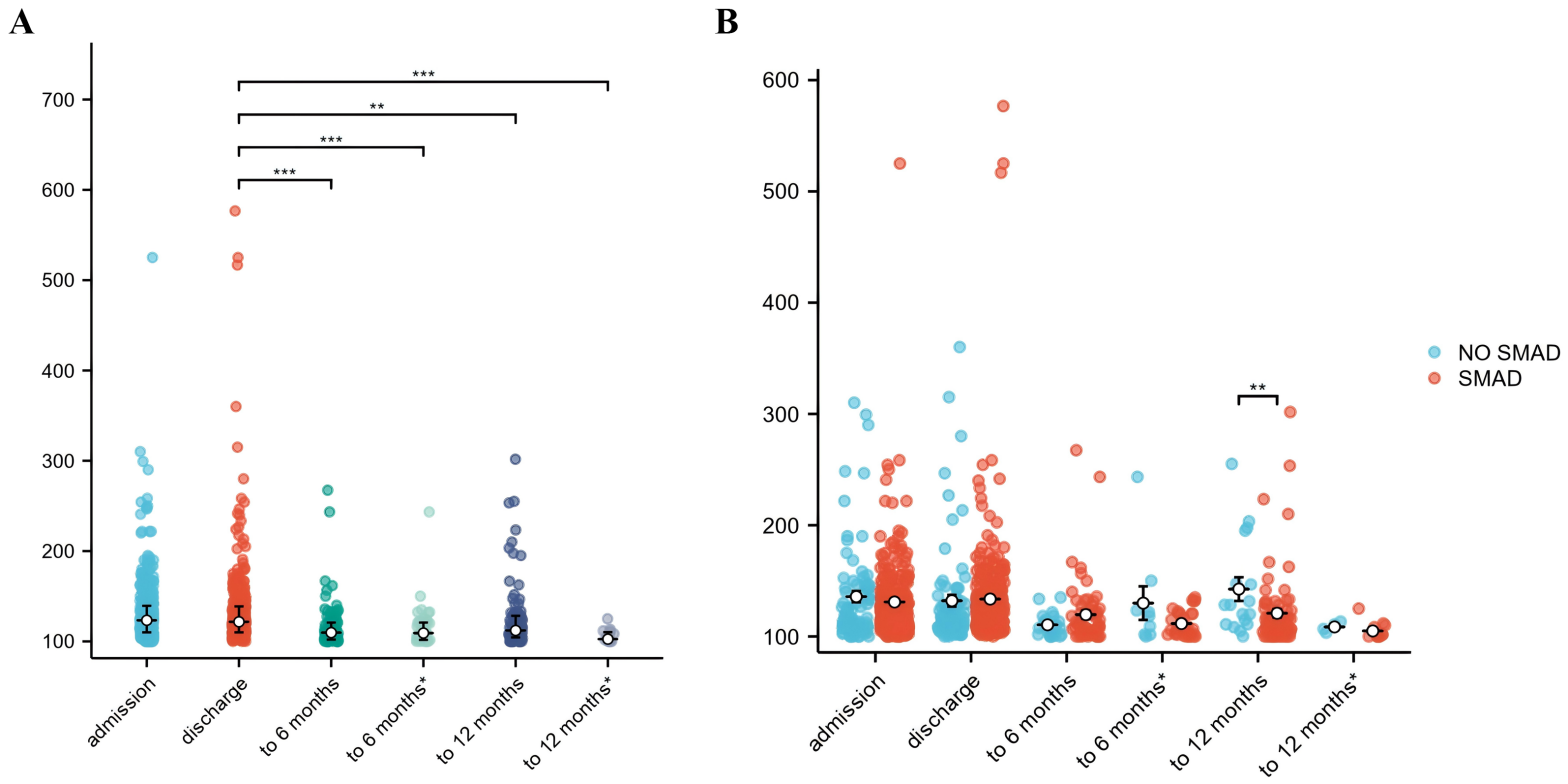
CT scores of PCPF patients. **(A)** All patients. **(B)** Group according to SMAD treatment. To 6 months: CT scores of all follow-up patients from discharge to 6 months after discharge. To 6 months*: CT scores of patients who were not readmitted from discharge to 6 months after discharge. To 12 months: CT scores of all follow-up patients from 6 months to 12 months after discharge. To 12 months*: CT scores of patients who were not readmitted from 6 months to 12 months after discharge. ***: *p* < 0.0001. **: *p* < 0.001. PCPF, post-COVID-19 pulmonary fibrosis; SMAD, small-molecule antiviral drug; CT, computed tomography.

The majority of patients reported that their CT had improved after treatment. The median CT score decreased from 123.33 (110.00–139.38) to 121.66 (110.00–138.75), but the difference was not statistically significant. Notably, compared with the CT score at discharge, the CT score at the 6-month and 12-month follow-ups was significantly lower, regardless of whether the CT scores of the readmitted patients were included (*p* < 0.001) (Figure 2A; Supplementary Table 2). However, no significant differences were observed between the 6-month and 12-month follow-up CT scores (Supplementary Figure 2A).

A comparison of the results revealed that there was no significant difference between the NO-SMAD and SMAD groups in terms of CT scores from admission to discharge, even at the 6-month follow-up (Supplementary Figure 3B; Supplementary Table 2). Notably, the CT scores at the 6- to 12-month follow-up in the NO-SMAD group, with a median of 128.33 (110.83–147.50), were significantly greater than those in the SMAD group [110.83 (103.33–123.33)] (*p* = 0.009) (Figure 2B; Supplementary Table 2).

### Outcomes and SMAD treatment

The median length of hospital stay for all the PCPF patients was 15 (11–21) days. Sixteen (4.30%) patients died in the hospital. A total of 132 of the 266 survivors were readmitted to the hospital for a median of 77 (23–204) days after discharge. An additional nineteen survivors died within 1 year following discharge. Notably, a total mortality rate of 12.41% (35/282) was observed, with a median time to death of 28 (0–184) days postdischarge, among those with observational outcomes throughout the study period. Patients who received prompt SMAD treatment had lower in-hospital mortality (8.86% vs. 3.07%, *p* = 0.024) and lower readmission rates (63.64% vs. 45.97%, *p* = 0.020) than did those in the NO-SMAD group. There was no substantial variability in 1-year death or event-days between the NO-SMAD group and the SMAD group (*p* > 0.05) (Table 1).

Further analysis of the outcome revealed more insights into the influence of SMADs. The SMAD group had a lower risk of in-hospital mortality (OR = 0.33, 95% CI: 0.12 to 0.90, *p* = 0.031) and a lower 1-year readmission rate (OR = 0.49, 95% CI: 0.26 to 0.9, *p* = 0.021). This association remained statistically significant even after adjusting for age and gender (Model 2, Hospital death OR = 0.31, 95% CI: 0.11, 0.86, *p* = 0.024; One-year readmitted OR = 0.49, 95% CI: 0.26, 0.90, *p* = 0.022). However, when a linear regression model was fitted, SMAD treatment was not significantly related to the number of days on which these outcomes occurred (Table 2).

**Table 2:** The relationship between SMAD and outcome.

Outcome	Events	*P*^b^ value	Event-days	*P*^c^ value
	OR (95%CI)		*β* (95%CI)	
Crude				
Hospital death	0.33 (0.12, 0.90)	0.031	−0.70 (−4.60, 3.21)	0.726
One-year readmitted	0.49 (0.26, 0.90)	0.021	−2.09 (−42.74, 38.56)	0.920
One-year death^a^	0.49 (0.23, 1.04)	0.065	0.58 (−80.90, 82.06)	0.989
Model 1				
Hospital death	0.31 (0.11, 0.87)	0.026	−0.37 (−4.26, 3.53)	0.854
One-year readmitted	0.49 (0.26, 0.90)	0.021	−4.26 (−44.94, 36.42)	0.838
One-year death^a^	0.47 (0.22, 1.02)	0.057	7.81 (−76.43, 92.05)	0.857
Model 2				
Hospital death	0.31 (0.11, 0.86)	0.024	−0.75 (−4.61, 3.10)	0.702
One-year readmitted	0.49 (0.26, 0.90)	0.022	−10.45 (−51.67, 30.76)	0.620
One-year death^a^	0.47 (0.22, 1.01)	0.052	4.56 (−101.18, 110.30)	0.933

Crude: No adjusted.

Model 1: Adjusted for age.

Model 2: Adjusted for age and gender.

a *N*= One-year death after discharge of follow-up patients+ hospital death. The percentage = 100% × number of One-year death/(Patients with One-year-follow-up + hospital death).

b Logistic regression analysis of the relationship between SMAD and events.

c Linear regression analysis of the relationship between SMAD and the duration of events.

SMAD, small molecule antiviral drug.

**Figure 3:**
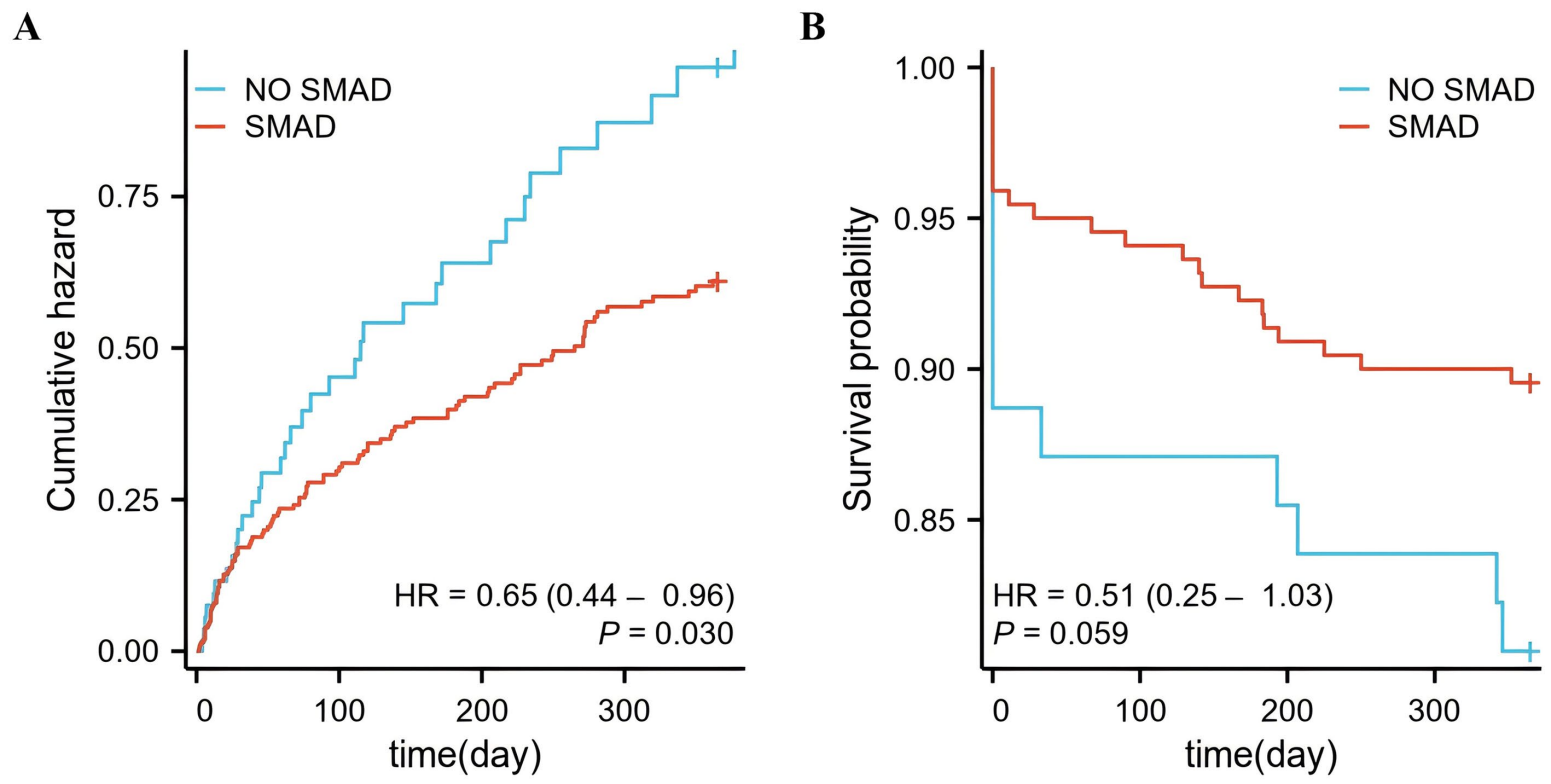
One-year readmission and mortality of PCPF patients. **(A)** One-year readmission of patients who were followed up. **(B)** One-year mortality of patients who were followed up, including in-hospital deaths. PCPF, post-COVID-19 pulmonary fibrosis; SMAD, small molecule antiviral drug.

Notably, a significant association between SMAD treatment and a reduced readmission rate (HR = 0.65, 95% CI: 0.44 to 0.96, *p* = 0.030) was detected by Cox regression analysis (Figure 3A). However, SMAD treatment was not significantly associated with 1-year mortality (HR = 0.51, 95% CI: 0.25 to 1.03, *p* = 0.059) (Figure 3B).

## Discussion

Our study revealed that pulmonary fibrosis induced by SARS-CoV-2 is not uncommon, affecting at least 3.17% of hospitalized patients with COVID-19. PCPF may have long-term implications; nearly 50% of patients under follow-up experienced critical illness and were readmitted within 1 year postdischarge, with some ultimately succumbing to respiratory complications. These basic findings are consistent with research showing that 4.8% (35/837) of COVID-19 survivors progressed to significant pulmonary fibrosis at 4 weeks after discharge (Myall et al., 2021).

In addition, we observed that the CT scores decreased at 6 months but increased at 12 months after discharge. Similarly, a previous study reported that the proportion of patients with post-COVID-19 respiratory symptoms slightly increased from 26% (313/1185) at the 6-month visit to 30% (380/1271) at the 12-month visit (*p* = 0.014) (Huang et al., 2021). The above evidence suggests that patients with PCPF require effective treatment to avoid these long-term problems.

Notably, the delayed and atypical nature of diagnostic indicators for PCPF may complicate treatment. The treatment of PCPF has focused mainly on the use of antifibrotic therapy and anti-inflammatory therapy. Antifibrotic drugs, such as nintedanib and pirfenidone, may be effective. However, the application of these drugs is limited by diagnostic delays, management challenges, and the high cost of the medications (George et al., 2020). To date, most relevant studies have been limited by small sample sizes (Kerget et al., 2023; Sansores et al., 2023), indicating the need for further investigations on the effectiveness of antifibrotic therapies for treating PCPF. Anti-inflammatory therapies can be initiated promptly during the acute infectious phase, but their use in clinical practice remains controversial. Early administration of corticosteroids was well tolerated and associated with rapid and significant improvement (Myall et al., 2021). Conversely, early treatment with dexamethasone did not appear to affect the incidence or clinical progression of PCPF, nor did it significantly influence clinical outcomes (Kooistra et al., 2023), which is similar to the results of this study (Supplementary Figure 4; Supplementary Table 3). Furthermore, there is an ambiguous distinction between the acute phase and fibrotic phase of COVID-19 infection (Solomon et al., 2021). However, research has shown that patients receiving high-dose corticosteroids during the fibrotic phase exhibited markedly reduced survival times; additionally, no significant differences in survival durations were observed across various dosage groups in the acute alveolitis phase (Chen et al., 2022). These findings complicate decisions regarding the optimal timing for initiating glucocorticoid therapy. Consequently, three critical dilemmas about PCPF treatment must be addressed: timeliness, convenience, and effectiveness. Therefore, SMADs may be the choice.

In accordance with established guidelines and policies, the application of SMADs during the acute phase of COVID-19 has proven to be both accessible and prompt. Fortunately, our study confirmed the effectiveness of SMADs for the treatment of PCPF via the correlation between SMAD treatment and low in-hospital mortality and readmission rates. Previous studies have shown a strong correlation between persistent reservoirs of SARS-CoV-2 and post-acute-COVID-19 symptoms. Chinese scholars reported that the detection of persistent SARS-CoV-2 RNA was significantly correlated with the occurrence of long COVID-19 symptoms in the convalescent population (Zuo et al., 2024). Relevant direct evidence has also been provided. For example, persistent reservoirs of SARS-CoV-2 (Yao et al., 2023) contribute to long-term dysfunction of taste papillae after COVID-19. The same phenomenon was observed in lung tissue. A study revealed SARS-CoV-2 RNA in 80% of lung tissue samples obtained from individuals up to 174 days after COVID-19, whose lung tissue samples were derived mainly from autopsies, explants, and surgical lung biopsies. In these lung samples, fibrotic changes were observed in 75% of the specimens (Roden et al., 2022). Therefore, the recovery, elimination of infectious virus, and development of immunity to acute nonretroviral RNA viruses do not necessarily indicate simultaneous elimination of the viral RNA (Griffin, 2022). In addition to the persistent reservoirs of SARS-CoV-2, related clinical manifestations in individuals infected with SARS-CoV-2 are caused not only by the direct toxicity of the virus in the acute phase but also by virus-triggered immunopathologic effects (Tanni et al., 2021; Lee et al., 2023). A randomized, controlled trial indicated that early antiviral therapy with SMADs could reduce the viral load quickly (Hammond et al., 2022). Data from the health care databases of the US Department of Veterans Affairs revealed that early treatment with SMADs was associated with a reduced risk of post-COVID-19 conditions at 180 days (Xie et al., 2023). These findings are consistent with our results, showing the significance of viral clearance in the acute phase of infection for impacting persistent reservoirs, alleviating the progression of PCPF.

Although SMADs have shown benefits in reducing readmission rates, their effect on one-year mortality remains limited. To further investigate this, we analyzed the causes of death in 35 patients with PCPF (Supplementary Table 4). Respiratory failure was the most frequent direct cause of death (20/35, 57.1%). including direct lung injury from severe SARS-CoV-2 infection which typically occurred during the acute or early recovery phase, and the subsequent severe pulmonary infections secondary to persistent architectural distortion of the lungs. The second most frequent cause of death was septic shock (8/35, 22.9%). This result suggests that patients may exhibit persistent immune dysfunction post-COVID-19. Additionally, corticosteroids or immunosuppressants used to manage fibrotic inflammation may further increase susceptibility to bacterial or fungal infections. It is noteworthy that a subset of patients ultimately succumbed to cardiovascular and thrombotic events. This observation underscores that the impact of the virus on the cardiovascular system cannot be overlooked (7/35, 20%). This risk may be amplified in elderly patients with pre-existing cardiovascular conditions. Therefore, even after recovery from the initial pulmonary infection, patients remain at significantly elevated risk for cardiovascular events, a risk beyond the protective scope of antiviral therapy alone.

Inevitably, there are several limitations because of the real-world, single-center study design. First, the incidence of PCPF in severe patients was 2.78% in this study. However, these results do not negate the high risk of pulmonary fibrosis in patients with severe disease (Tanni et al., 2021). This inaccuracy tied well with the strict inclusion criteria based on CT examinations. Owing to the particularity of the illness, patients in the ICU lacked multiple chest CT scans and were instead given single CT scans or multiple bedside X-ray examinations. Second, not all of the remaining patients completed chest CT examinations at this medical institution for reasons related to patients’ willingness to participate and the accessibility of follow-up visits in this real-world study. Additionally, pulmonary function tests were performed in very few patients, consequently, our study findings cannot reflect the functional pulmonary changes in PCPF patients. Third, the SMADs treatment was initiated during the acute phase in this study. However, the potential need for extended antiviral treatment into the fibrotic phase, as well as the optimal treatment duration, were not thoroughly explored in our research. We acknowledge that the viral persistent reservoir may act as a sustained antigenic stimulus, potentially driving chronic inflammation and fibrosis, a phenomenon observed with other viral infections. Whether sustained antiviral therapy is beneficial in this context remains an open question. Future studies are warranted to further investigate this issue. Finally, 90 survivors were lost to follow-up in this study. Fortunately, there was no significant difference in the 1-year follow-up rate between NO-SMAD group and the SMAD group (69.62% vs. 72.01%, *p* = 0.715), which did little affect the conclusion of the effect of SMADs on the long-term progression of PCPF patients. We conducted a comparative analysis of baseline characteristics between these lost-to-follow-up patients and those who completed follow-up (Supplementary Table 5). While most baseline variables showed no significant statistical differences between the two groups, the rate of glucocorticoid or immunosuppressive medication use was significantly higher among patients who remained in follow-up. Given the established role of immune dysregulation in the progression of PCPF, this observation raises the question of whether patients with pre-existing immune abnormalities are more susceptible to disease progression. Future prospective studies incorporating more rigorous immunophenotyping are warranted to further explore this hypothesis.

Despite these limitations, several advantages of our study need to be emphasized. The PCPF dataset included data from patients with acute SARS-CoV-2 infection who had reliable evidence and who were continuously observed on the basis of real-world data. This large sample size is beneficial for the generalizability of the study results. Furthermore, bacterial superinfection and mechanical ventilation are important risk factors during acute COVID-19 (Tanni et al., 2021). In this study, most PCPF patients were treated with antibiotics to control secondary infections (94.62%), and few patients were treated with invasive oxygen therapy (4.84%). These results effectively reduce the interference of the above risk factors. Finally, we are the first to confirm a significant association between SMAD treatment and 1-year readmission rates, providing a reliable explanation for the existing intractable treatment of PCPF.

## Conclusion

Our findings will hopefully inform future investigations on the characteristics and progression of pulmonary fibrosis following COVID-19 and identify potential effective treatments. Promisingly, with respect to both short-term and one-year prognosis, SMADs may confer benefits to PCPF patients as a convenient and readily available treatment.

## Data Availability

The raw data supporting the conclusions of this article will be made available by the authors, without undue reservation.
