# Supplementary material for: The effect and safety of small-molecule antiviral drugs on long prognosis of post-COVID-19 pulmonary fibrosis: a real-world study in China
Source: Front Microbiol. 2026 Jan 6;16:1733465. doi: 10.3389/fmicb.2025.1733465 (PMC12816351; doi:10.3389/fmicb.2025.1733465)
Supplement: Supplementary file 1 [file Data_Sheet_1.docx]

| Supplementary Table 1. Blood markers of systemic inflammation before and after treatment.(N=372) | | | | | | |
| --- | --- | --- | --- | --- | --- | --- |
| Variables | Before treatment | | After treatment | | P^b^ | P^c^ |
|  | Median(IQR) | N(%)^a^ | Median(IQR) | N(%)^a^ |  |  |
| Lymphocytes (10^9^/L) | 0.76 (0.53-1.16) | 267 (71.77%) | 1.00 (0.66-1.42) | 205 (55.11%) | <0.001 | <0.001 |
| CRP (mg/L) | 31.14 (10.84-72.72) | 282 (75.81%) | 9.28 (2.33-25.10) | 188 (53.26%) | <0.001 | <0.001 |
| ^a^: Abnormal cases, Lymphocytes<1.1×10^9^/L, CRP>8mg/L.  P^b^: Median (IQR) of Before treatment vs Median (IQR) of After treatment.  P^c^: N (%) of Before treatment vs N (%) of After treatment.  CRP: C-reactive protein. | | | | | | |

| Supplementary Table 2. CT scores of PCPF patients. | | | | |
| --- | --- | --- | --- | --- |
| CT scores | **All PCPF** | **NO SMAD** | **SMAD** | **P-value** |
|  | N=372 | N=79 | N=293 |  |
| admission | 123.33 (110.00-139.38) | 119.17 (107.91-145.00) | 124.16 (110.83-138.33) | 0.579 |
| discharge | 121.66 (110.00-138.75) | 116.67 (108.31-132.08) | 123.30 (110.50-140.00) | 0.072 |
| to 6 months | 109.67 (102.00-120.83) | 109.42 (101.70-114.58) | 110.00 (103.33-122.91) | 0.305 |
| to 6 months* | 108.25 (101.68-120.71) | 107.33 (100.00-120.33) | 109.17 (102.00-121.83) | 0.490 |
| to 12 months | 112.08 (104.37-128.33) | 128.33 (110.83-147.50) | 110.83 (103.33-123.33) | 0.009 |
| to 12 months* | 102.52 (100.00-110.21) | 100.00 (100.00-100.00) | 103.33 (100.41-110.42) | 0.226 |
| to 6 months: CT scores of all follow-up patients from discharge to 6 months after discharge.  to 6 months*: CT scores of patients NOT readmitted from discharge to 6 months after discharge.  to 12 months: CT scores of all follow-up patients from 6 months to 12 months after discharge.  to 12 months*: CT scores of patients NOT readmitted from 6 months to 12 months after discharge.  SMAD: small molecule antiviral drugs. | | | | |

| Supplementary Table 3. Association between all medications and outcomes analyzed by Logistic regression | | | |
| --- | --- | --- | --- |
|  | Hpspital death | One-year readmitted | One-year death^a^ |
|  | OR (95%CI) | OR (95%CI) | OR (95%CI) |
| SMAD | 0.33 (0.12, 0.90)* | 0.49 (0.26, 0.90)* | 0.49 (0.23, 1.04) |
| Glucocorticoid | 0.58 (0.16, 2.12) | 0.36 (0.16, 0.81) * | 0.62 (0.24, 1.62) |
| Immunoglobulin | 2.82 (1.00, 7.81) | 1.31 (0.74, 2.30) | 1.43 (0.66, 3.09) |
| Oxygen therapy | 2.34 (0.30, 18.10) | 1.17 (0.60, 2.28) | 2.00 (0.58, 6.86) |
| One-year death^a^: One-year death after discharge of follow-up patients+ hospital death. The percentage= 100%× number of One year death/(Patients with One-year-follow-up+ hospital death).  P: Logistic regression analysis of the relationship between medicine and events.**: <0.001; *: <0.05 | | | |

| Supplementary Table 4. Underlying causes of death in the 35 deceased patients. | | |
| --- | --- | --- |
| Gender | Age(year) | Underlying Cause of Death |
| Male | 70 | Respiratory Failure |
| Male | 87 | Respiratory Failure |
| Male | 73 | Respiratory Failure |
| Male | 75 | Respiratory Failure |
| Male | 74 | Respiratory Failure |
| Male | 82 | Respiratory Failure |
| Female | 57 | Respiratory Failure |
| Female | 27 | Septic Shock |
| Male | 92 | Respiratory Failure |
| Male | 89 | Septic Shock |
| Male | 87 | Cardiogenic Shock |
| Male | 76 | Pulmonary Embolism |
| Male | 47 | Septic Shock |
| Female | 82 | Respiratory Failure |
| Male | 50 | Respiratory Failure |
| Male | 94 | Respiratory Failure |
| Female | 44 | Septic Shock |
| Female | 74 | Respiratory Failure |
| Female | 68 | Cardiogenic Shock |
| Female | 54 | Septic Shock |
| Male | 65 | Respiratory Failure |
| Male | 46 | Cardiac failure |
| Male | 74 | Respiratory Failure |
| Male | 59 | Septic Shock |
| Male | 84 | Respiratory Failure |
| Female | 86 | Cardiogenic Shock |
| Male | 58 | Sudden Cardiac Death |
| Female | 87 | Respiratory Failure |
| Female | 84 | Respiratory Failure |
| Male | 50 | Septic Shock |
| Male | 80 | Respiratory Failure |
| Male | 60 | Cardiogenic Shock |
| Male | 56 | Septic Shock |
| Male | 86 | Respiratory Failure |
| Male | 70 | Respiratory Failure |

| Supplementary Table 5**.** Characteristics of Patients Lost to Follow-Up Compared with Completers. | | | |
| --- | --- | --- | --- |
| **Variables** | **Lost to Follow-up** | **Follow-up** | **P-value** |
|  | N=90 | N=282 |  |
| Age (year) | 76 (66-82) | 69 (60-79) | 0.095 |
| **Gender** |  |  |  |
| Female | 27 (30.00%) | 102 (36.17%) | 0.284 |
| Male | 63 (70.00%) | 180 (63.83%) |  |
| BMI | 22.49 (20.64-24.49) | 22.93 (20.84-24.96) | 0.178 |
| Current smoker | 9 (10.00%) | 32 (11.35%) | 0.722 |
| Current drinker | 4 (4.44%) | 24 (8.51%) | 0.203 |
| **Comorbidity** |  |  |  |
| Hypertension | 42 (46.67%) | 152 (53.90%) | 0.232 |
| CVD | 22 (24.44%) | 57 (20.21%) | 0.393 |
| COPD | 15 (16.67%) | 39 (13.83%) | 0.506 |
| TB | 7 (7.78%) | 11 (3.90%) | 0.136 |
| Diabetes | 23 (25.56%) | 85 (30.14%) | 0.404 |
| Hyperlipidemia | 2 (2.22%) | 16 (5.67%) | 0.184 |
| CLD | 8 (8.89%) | 33 (11.70%) | 0.458 |
| CKD | 13 (14.44%) | 55 (19.50%) | 0.280 |
| Rheumatic | 6 (6.67%) | 30 (10.64%) | 0.267 |
| Anemia | 21 (23.33%) | 64 (22.70%) | 0.900 |
| Athma | 0 (0.00%) | 2 (0.71%) | 0.423 |
| Medication^a^ | 2 (2.22%) | 64 (22.70%) | <0.001 |
| Severe | 39 (43.33%) | 98 (34.75%) | 0.142 |
| **Vital signs** |  |  |  |
| Temperature (℃) | 36.7 (36.3-37.1) | 36.6 (36.4-37.1) | 0.902 |
| SBP (mmHg) | 127 (115-138) | 127 (115-140) | 0.587 |
| DBP (mmHg) | 73 (66-82) | 75 (67-83) | 0.430 |
| Pulse (per min) | 85 (79-97) | 89 (78-100) | 0.212 |
| **Symptoms of COVID-19** |  |  |  |
| NO fever | 21 (23.33%) | 57 (20.21%) | 0.567 |
| Low fever (37.2-38.5) | 33 (36.67%) | 121 (42.91%) |  |
| High fever (>38.5) | 36 (40.00%) | 104 (36.88%) |  |
| Fatigue | 27 (30.00%) | 110 (39.01%) | 0.123 |
| Cough | 85 (94.44%) | 258 (91.49%) | 0.363 |
| Sputum | 83 (92.22%) | 243 (86.17%) | 0.129 |
| Nasal congestion / runny nose | 2 (2.22%) | 11 (3.90%) | 0.45 |
| Sore throat | 5 (5.56%) | 30 (10.64%) | 0.15 |
| Hypogeusia | 0 (0.00%) | 1 (0.35%) | 0.572 |
| Digestive | 4 (4.44%) | 16 (5.67%) | 0.653 |
| Myalgia | 10 (11.11%) | 24 (8.51%) | 0.456 |
| Chest distress | 46 (51.11%) | 110 (39.01%) | 0.115 |
| Dyspnea | 18 (20.00%) | 68 (24.11%) | 0.671 |
| **Oxygen therapy** (>24 h) |  |  |  |
| NO oxygen therapy | 26 (28.89%) | 104 (36.88%) | 0.127 |
| Non invasive oxygen therapy | 46 (51.11%) | 110 (39.01%) |  |
| Invasive oxygen therapy | 18 (20.00%) | 68 (24.11%) |  |
| **Therapy** |  |  |  |
| SMAD | 73 (81.11%) | 220 (78.01%) | 0.532 |
| Glucocorticoid | 79 (87.78%) | 248 (87.94%) | 0.967 |
| Immunoglobulin | 19 (21.11%) | 65 (23.05%) | 0.834 |
| Antibiotic | 88 (97.78%) | 264 (93.62%) | 0.128 |
| Hospital stay (day) | 15 (11-19) | 15 (11-21) | 0.421 |
| Medication^a^: Immunosuppressant or corticosteroids.  PCPF: post-COVID-19 pulmonary fibrosis; COVID-19: coronavirus disease 2019; SMAD: small molecule antiviral drug; BMI: body mass index; ILD: interstitial lung disease; CVD: cardiovascular disease; COPD: chronic obstructive pulmonary disease; TB: tuberculosis; CLD: chronic liver disease; CKD: chronic kidney disease. | | | |


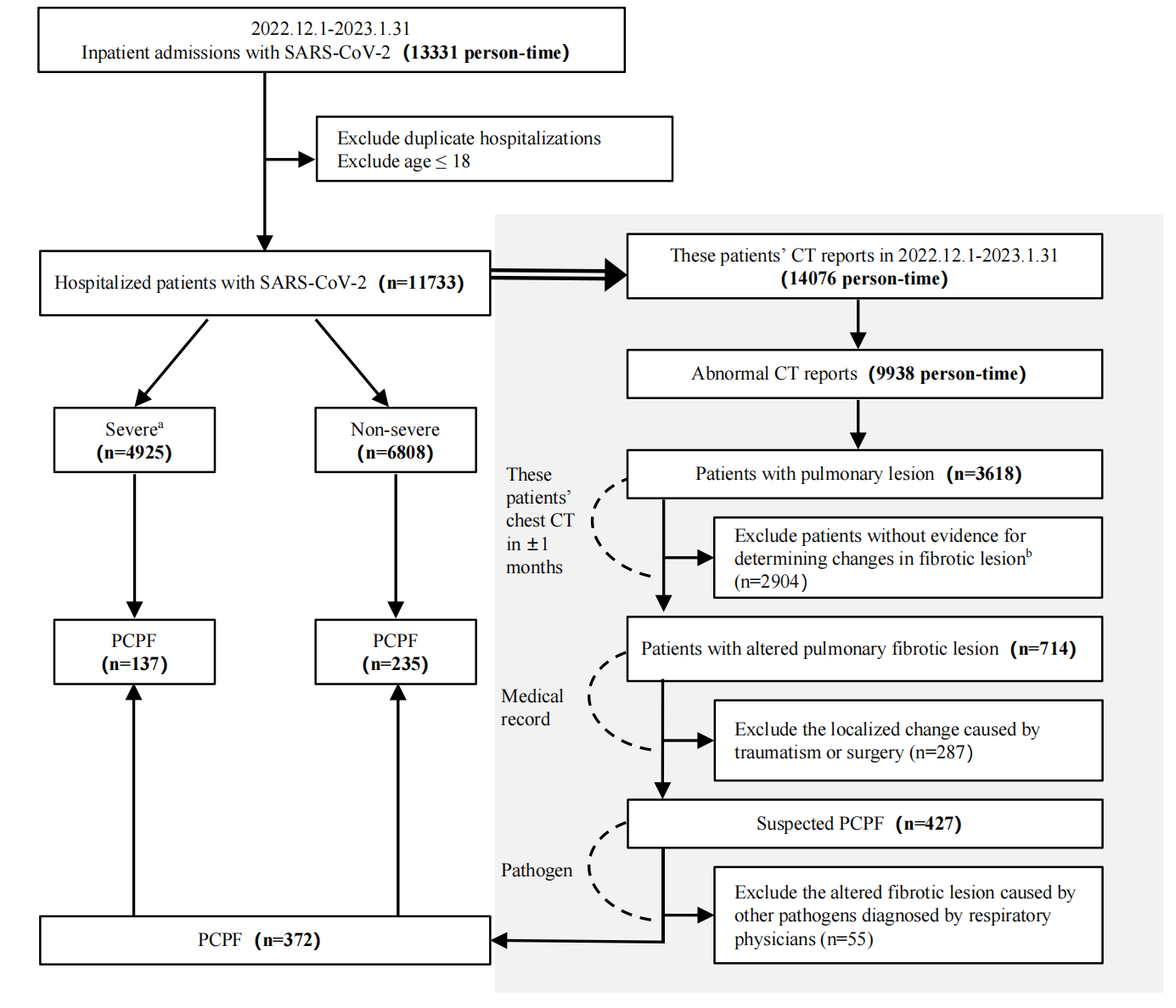


**Supplementary Figure 1. Flowchart of the study population.**

a Severe：Patients admitted to the ICU with respiratory failure or with shock

b Evidence for determining changes in fibrotic lesions: 1) aggravated fibrotic lesions during hospitalization compared with those observed one month prior to admission; 2) progressive aggravation of fibrotic lesions during hospitalization; and 3) increased severity of fibrotic lesions within one month after discharge compared with those noted during hospitalization.

SARS-CoV-2: severe acute respiratory syndrome coronavirus 2; CT: computed tomography; PCPF: post COVID-19 pulmonary fibrosis; COVID-19: coronavirus disease 2019.


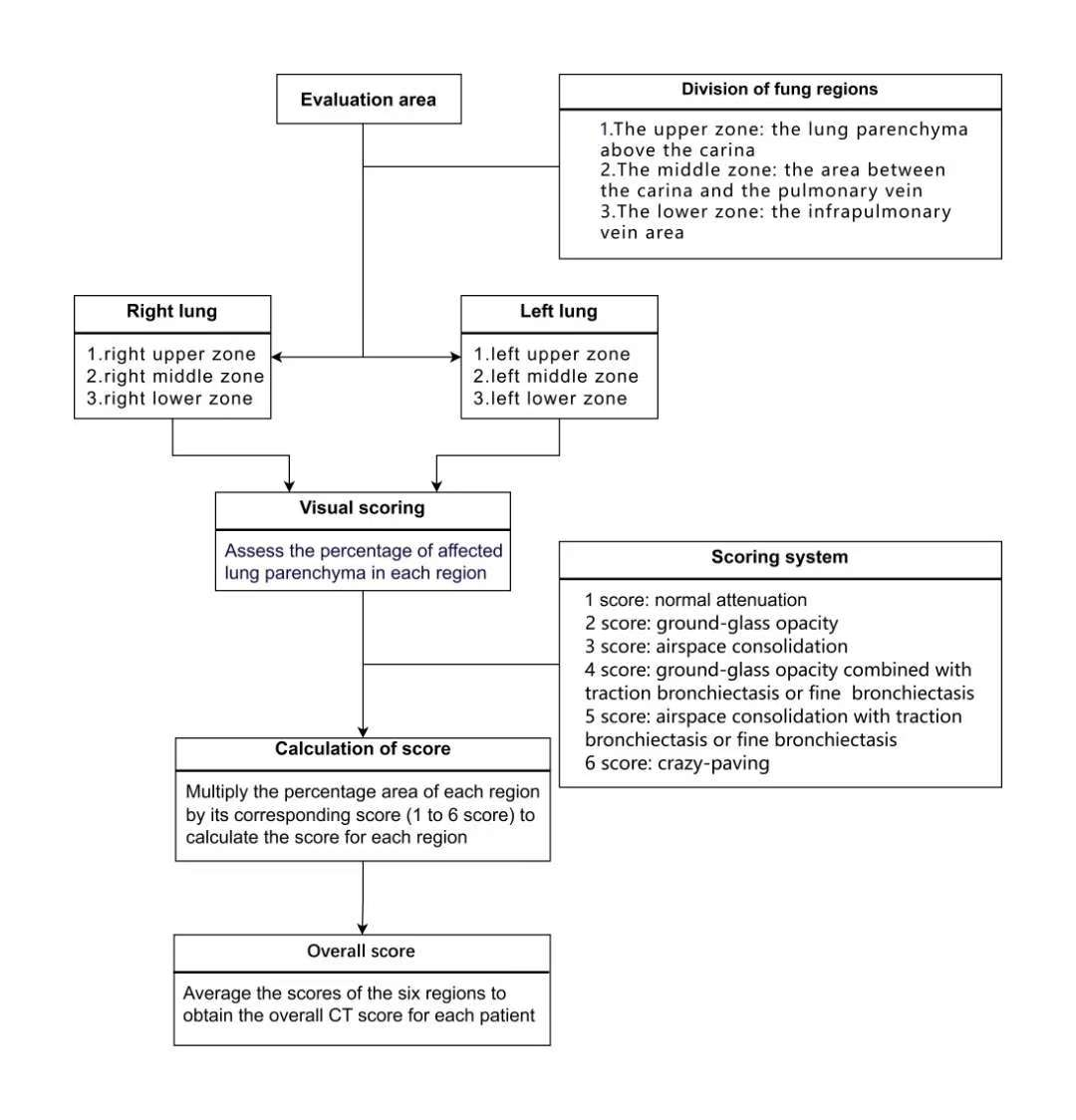


**Supplementary Figure 2. Ichikado CT scores of PCPF patients.**

CT: computed tomography; PCPF: post COVID-19 pulmonary fibrosis; COVID-19: coronavirus disease

2019.


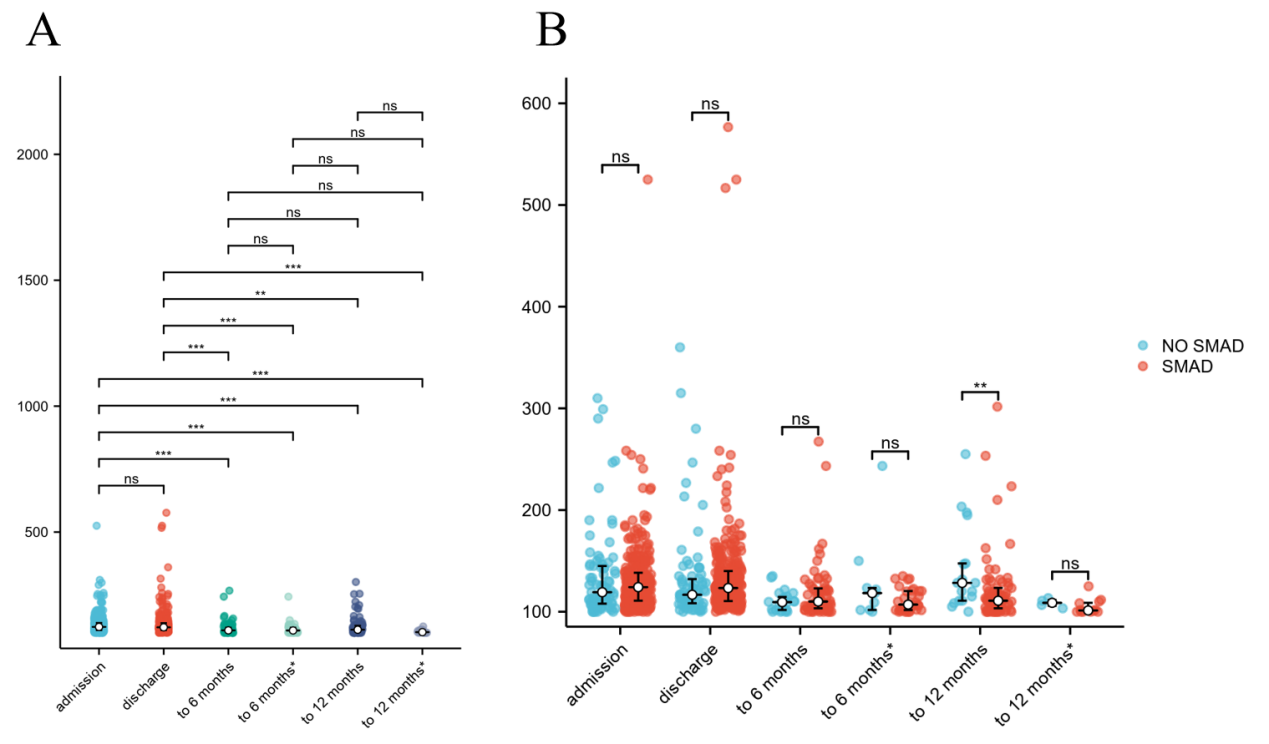


**Supplementary Figure 3. CT scores of PCPF patients.** A) All patients. B) Group according to SMAD

treatment.

to 6 months: CT scores of all follow-up patients from discharge to 6 months after discharge.

to 6 months*: CT scores of patients who were not readmitted from discharge to 6 months after discharge.

to 12 months: CT scores of all follow-up patients from 6 months to 12 months after discharge.

to 12 months*: CT scores of patients who were not readmitted from 6 months to 12 months after discharge.

***: P<0.0001; **: P<0.001; *: P<0.05; ns: P>0.05

SMAD: small molecule antiviral drug; CT: computed tomography; PCPF: post COVID-19 pulmonary

fibrosis; COVID-19: coronavirus disease 2019.


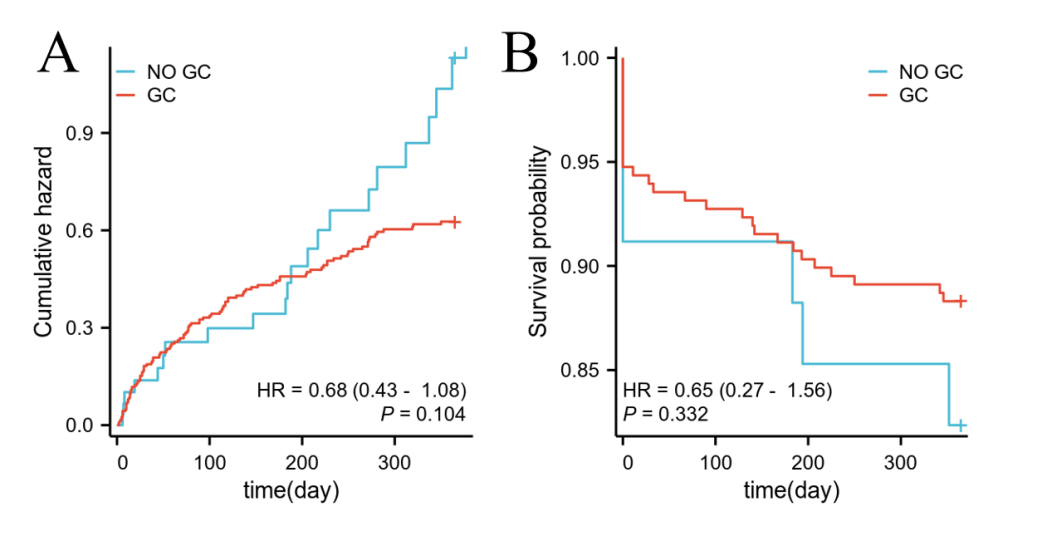


**Supplementary Figure 4. One-year readmission and mortality of PCPF patients.** A) One-year readmission of patients who were followed up. B) One-year mortality of patients who were followed up, including in-hospital deaths.

GC: glucocorticoid; PCPF: post COVID-19 pulmonary fibrosis; COVID-19: coronavirus disease 2019.
